# The SNPs of mitochondrial DNA displacement loop region and mitochondrial DNA copy number associated with risk of polymyositis and dermatomyositis

**DOI:** 10.1038/s41598-022-09943-x

**Published:** 2022-04-07

**Authors:** Yufei Zhao, Chenxing Peng, Ruixue Lai, Jingjing Zhang, Xiaoyun Zhang, Zhanjun Guo

**Affiliations:** 1 Department of Immunology and Rheumatology, The Fourth Hospital of Hebei Medical University, 12 Jiankang Road, Shijiazhuang, 050011 Hebei People’s Republic of China; 2 Department of Immunology and Rheumatology, The Second Hospital of Hebei Medical University, Shijiazhuang, 050000 Hebei People’s Republic of China

**Keywords:** Mitochondria, Idiopathic inflammatory myopathies, Interleukins, Predictive markers

## Abstract

Oxidative damage-induced mitochondrial dysfunction may activate muscle catabolism and autophagy pathways to initiate muscle weakening in idiopathic inflammatory myopathies (IIMs). In this study, Single nucleotide polymorphisms (SNPs) in the mitochondrial displacement loop (D-loop) and mitochondrial DNA (mtDNA) copy number were assessed and their association with the risk of polymyositis and dermatomyositis (PM/DM) was evaluated. Excessive D-loop SNPs (8.779 ± 1.912 vs. 7.972 ± 1.903, *p* = 0.004) correlated positively with mtDNA copy number (0.602 ± 0.457 vs. 0.300 ± 0.118, *p* < 0.001). Compared with that of the controls, the mtDNA of PM/DM patients showed D-loop SNP accumulation. In addition, the distribution frequencies of 16304C (*p* = 0.047) and 16519C (*p* = 0.043) were significantly higher in the patients with PM/DM. Subsequent analysis showed that reactive oxygen species (ROS) generation was increased in PM/DM patients compared with that in the controls (18,477.756 ± 13,574.916 vs. 14,484.191 ± 5703.097, *p* = 0.012). Further analysis showed that the PM/DM risk-related allele 16304C was significantly associated with lower IL-4 levels (*p* = 0.021), while 16519C had a trend to be associated with higher IL-2 expression (*p* = 0.064). The allele 16519C was associated with a positive antinuclear antibody (ANA) status in PM/DM patients (*p* = 0.011). Our findings suggest that mitochondrial D-loop SNPs could be potential biomarkers for PM/DM risk and these SNPs associated with cytokine expression may be involved in the development of PM/DM. Further, mtDNA copy number-mediated mitochondrial dysfunction may precede the onset of PM/DM.

## Introduction

Idiopathic inflammatory myopathies (IIMs) are a heterogeneous group of autoimmune diseases that are characterized by chronic muscle inflammation. The reported annual incidence of IIMs varies from 1.16 to 19 per million^1^. Polymyositis (PM) and dermatomyositis (DM) are the most common IIM subtypes that frequently affect the skin, lungs, heart, joints, and gastrointestinal tract, with multiple system involvement, leading to a poor prognosis. Geographical disparities exist in the incidence of PM and DM; however, the incidence of DM is dramatically higher in China^2,3^. Available evidence suggests that a combination of immune and non-immune factors is involved in the pathogenesis of PM/DM^4,5^. Oxidative damage-induced mitochondrial dysfunction may activate muscle catabolism and autophagy pathways to initiate muscle weakening in IIMs^6^. However, the exact mechanism underlying this disease remains unknown.

Human mitochondrial DNA (mtDNA) is a 16-kb closed, double-stranded circular molecule that is particularly susceptible to mutations owing to the high rate of reactive oxygen species (ROS) generation, lack of protective histones, and relatively inefficient DNA repair system^7,8^. The displacement loop (D-loop) is a triple-stranded structure located in the main non-coding region, which contains the elements that regulate replication and expression of mtDNA^9^. The mutations in this region modulate mtDNA expression, mitochondrial function, oxidative phosphorylation, and ATP production^10^. The predictive value of single nucleotide polymorphisms (SNPs) in the mtDNA D-loop has been reported in various diseases, including colon cancer, rheumatoid arthritis, and systemic lupus erythematosus^11–13^. Additionally, the mtDNA copy number, which reflects gene-environment interactions between unknown hereditary factors and oxidative stress exposure, has been proven to be a risk predictor of breast cancer, cardiovascular diseases, and neurodegenerative diseases^14–16^. However, the predictive value of SNPs in the mtDNA D-loop region and mtDNA copy number in PM/DM is not clear.

Oxidative stress and cytokines are widely involved in the pathophysiology of PM/DM and are believed to be crucial for the regulation mitochondrial function^6,17^. Increasing evidence demonstrates that ROS plays a key role in skeletal muscle weakness in PM/DM by mediating mitochondrial dysfunction, depressed force generation, and activation of muscle catabolic and autophagy pathways^6^. Mitochondrial metabolism is implicated in cytokine induction by regulating T cell activation, differentiation, and formation^18^. Cytokines play a substantial role in the development of PM/DM, not only through their pro and anti-inflammatory effects but also by direct action on muscle tissue^19^.

In this study, we performed mtDNA assessment in patients with PM/DM to evaluate its association with the development of PM/DM, with particular focus on ROS and cytokine involvement.

## Results

A total of 86 PM/DM patients and 110 controls were included in this study. The clinical characteristics of the cases and controls are shown in Table 1, with no statistical differences in age and sex between the two groups.

**Table 1:** Clinical characteristics of PM/DM patients and controls.

Group	PM/DM patients (n = 86)	Controls (n = 110)	T value	χ^2^	*p*
Age (year)	52.92 ± 14.24	55.08 ± 10.66	−1.175	–	0.242
Gender (M/F)	17/69	20/90	−	0.079	0.778
PM/DM	10/76				
**Manifestations**					
Muscle pain	34	–			
Muscle weakness	51	–			
Heliotrope rash	48	–			
Gottron’s sign	40	–			
Shawl and V-sign	39	–			
ILD	48	–			
Dysphagia	12	–			
ANA ( +)	68	–			
Jo-1 ( +)	6	–			
CK (U/L)	1797.720 ± 3079.345	–			
LDH (U/L)	562.436 ± 869.004	–			
ESR (mm/h)	29.744 ± 23.014	–			
CRP (mg/L)	10.962 ± 22.224	–			

*PM* polymyositis, *DM* dermatomyositis, *ILD* interstitial lung disease, *ANA* antinuclear antibody, *χ*^2^ Chi-square.

**Figure 1:**
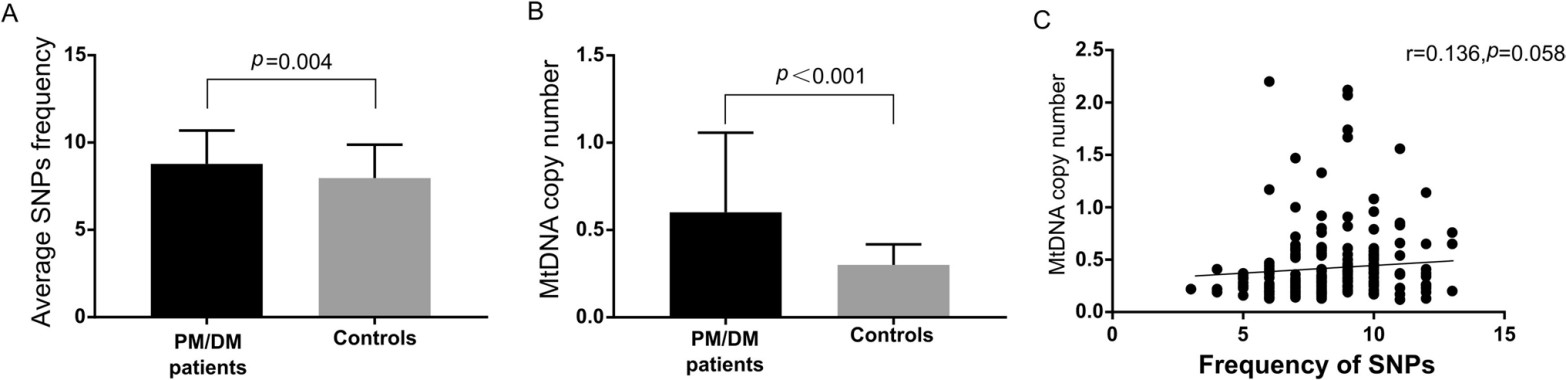
Average SNPs frequency, mtDNA copy numbers and their correlations in PM/DM patients and controls. **(A)** The average frequency of SNPs in PM/DM patients and controls. **(B)** MtDNA copy number in PM/DM patients and controls. **(C)** Correlation between Frequency of SNPs and mtDNA copy number. *PM* polymyositis, *DM* dermatomyositis, *SNP* single nucleotide polymorphisms, *MtDNA* mitochondrial DNA.

SNPs were detected at 116 sites in the mitochondrial D-loop of patients with PM/DM. The average frequency of SNPs in each PM/DM patient was significantly greater than that in controls (8.779 ± 1.912 vs. 7.972 ± 1.903, *p* = 0.004, Fig. 1A), demonstrating that D-loop SNPs accumulate more frequently in PM/DM patients. Twenty-two SNPs with minor allele frequencies greater than 5% in patients with PM/DM or controls were used for risk analysis (Table 2). The alleles 16304C (16.3% vs. 7.3%, *p* = 0.047) and 16519C (48.8% vs. 34.5%, *p* = 0.043) in the mitochondrial D-loop were identified for their association with PM/DM risk by comparing PM/DM patients with healthy controls. The PM/DM risk-associated allele 16519C was also associated with the antinuclear antibody (ANA) status (90.5% vs. 68.2%, *p* = 0.011, Table 3). Additionally, the mtDNA copy number in PM/DM patients was significantly higher than that in controls (0.602 ± 0.457 vs. 0.300 ± 0.118, *p* < 0.001, Fig. 1B). The relationship between mtDNA copy number and SNP frequency was subsequently evaluated by the Pearson’s correlation test, although no statistical significance was obtained, and a positive correlation trend was found (r = 0.136, *p* = 0.058, Fig. 1C). This result implies that mitochondrial D-loop SNPs and increased mtDNA copy number may be associated with PM/DM.

**Table 2:** Frequency distribution for mitochondrial D-loop single nucleotide polymorphisms between PM/DM patients and controls.

Nucleotide	PM/DM patients (n = 86)	Controls (n = 110)	*χ*^2^	*p*	OR
16266C/T	79/7 (91.9%/8.1%)	103/7 (93.6%/6.4%)	0.230	0.632	0.767
16291C/T	79/7 (91.9%/8.1%)	99/11 (90.0%/10.0%)	0.200	0.654	1.254
16298T/C	79/7 (91.9%/8.1%)	101/9 (91.8%/8.2%)	0.000	0.991	1.006
16304T/C	72/14 (83.7%/16.3%)	102/8 (92.7%/7.3%)	3.929	0.047	0.403
16311T/C	77/9 (89.5%/10.5%)	102/8 (92.7%/7.3%)	0.621	0.431	0.671
16316A/G	84/2 (97.7%/2.3%)	104/6 (94.5%/5.5%)	0.540	0.462	2.423
16319G/A	78/8 (90.7%/9.3%)	92/18 (83.6%/16.4%)	2.092	0.148	1.908
16362T/C	46/40 (53.5%/46.5%)	62/48 (56.4%/43.6%)	0.161	0.688	0.890
16519T/C	44/42 (51.2%/48.8%)	72/38 (65.5%/34.5%)	4.081	0.043	0.553
103G/A	81/5 (94.2%/5.8%)	106/4 (96.4%/3.6%)	0.144	0.705	0.611
146T/C	72/14 (86.0%/14.0%)	97/13 (88.2%/11.8%)	0.809	0.369	0.689
150C/T	66/20 (76.7%/23.3%)	92/18 (83.6%/16.4%)	1.467	0.226	0.646
151C/T	80/6 (93.0%/7.0%)	109/1 (99.1%/0.9%)	3.548	0.060	0.122
152T/C	68/18 (79.1%/20.9%)	86/24 (78.2%/21.8%)	0.023	0.880	1.054
195T/C	76/10 (88.4%/11.6%)	99/11 (90.0%/10.0%)	0.134	0.715	0.844
199T/C	84/2 (97.7%/2.3%)	103/7 (93.6%/6.4%)	0.993	0.319	2.854
204T/C	79/7 (91.9%/8.1%)	104/6 (94.5%/5.5%)	0.562	0.454	0.651
207G/A	79/7 (91.9%/8.1%)	105/5 (95.5%/4.5%)	1.085	0.298	0.537
235A/G	79/7 (91.9%/8.1%)	98/12 (89.1%/10.9%)	0.423	0.515	1.382
249A/del	65/21 (91.9%/8.1%)	88/22 (80.0%/20.0%)	0.550	0.458	0.774
489T/C	43/43 (50.0%/50.0%)	56/54 (50.1%/49.9%)	0.016	0.899	0.964
523-524AC/del	61/25 (70.9%/29.1%)	64/46 (58.2%/41.8%)	3.395	0.065	1.754

*PM* polymyositis, *DM* dermatomyositis, *χ*^2^ Chi-square, *OR* odd ratio.

*Including C and CC insertion.

**Table 3:** Allele 16519 associated with positive expression of ANA in PM/DM patients.

Characteristic	16519C	16519T	*χ*^2^	*p*	OR
ANA			6.454	0.011	4.433
( +)	38 (90.5%)	30 (68.2%)			
(–)	4 (9.5%)	14 (31.8%)			

*PM* polymyositis, *DM* dermatomyositis; *χ*^2^ Chi-square, *OR* odds ratio, *ANA* antinuclear antibody.

The predictive value of mtDNA copy number and SNPs in the mitochondrial D-loop were further investigated in the subgroup analysis. As shown in Fig. S1, the mtDNA copy number was significantly higher in 10 PM patients (0.876 ± 0.652 vs. 0.300 ± 0.118, *p* = 0.021) and 76 DM patients (0.566 ± 0.417 vs. 0.300 ± 0.118, *p* < 0.001). Excessive SNP accumulation was observed in DM patients compared with that in controls (8.803 ± 1.890 vs. 7.792 ± 1.903, *p* = 0.004, Fig. S1D), but this was not the case in PM patients (8.600 ± 2.171 vs. 7.792 ± 1.903, *p* = 0.326, Fig. S1C). The allele 16304C (17.1% vs. 7.3%, *p* = 0.037, Table S1) was significantly associated with DM risk, while the allele 16519C only showed a trend for DM risk (47.4% vs. 34.5%, *p* = 0.079, Table S1). However, the association between D-loop SNPs and PM risk was not identified in patients with PM, likely owing to the small sample size of 10.

**Figure 2:**
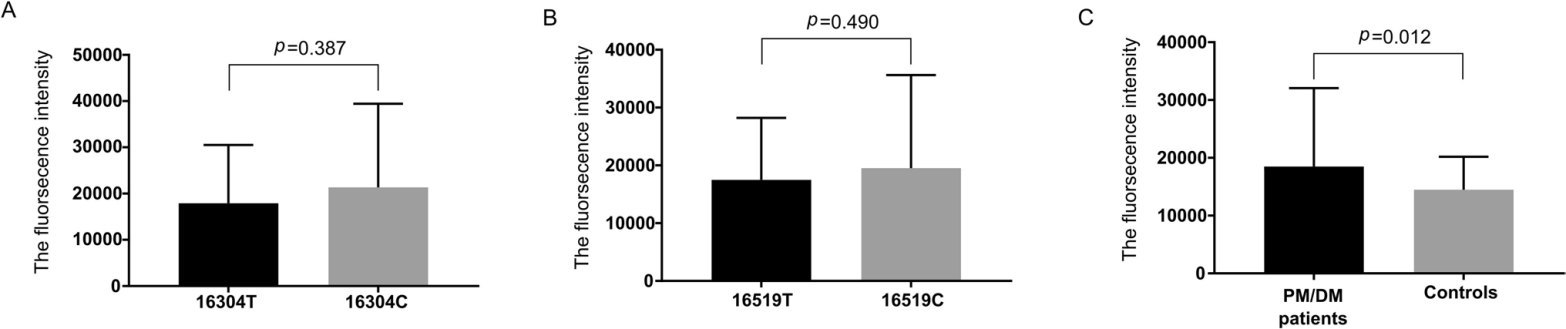
Reactive oxygen species level in PM/DM patients. **(A)** The ROS levels in 16304T and 16304C patients. **(B)** The ROS levels in 16519T and 16519C patients. **(C)** The ROS levels in PM/DM patients and controls. *PM* polymyositis, *DM* dermatomyositis, *SNP* single nucleotide polymorphisms.

Subsequent analysis of PM/DM-susceptible SNPs and ROS levels (Fig. 2) did not reveal a significant association (16,304: T 17,915.778 ± 12,604.843 vs. C 21,367.929 ± 18,066.864, *p* = 0.387; 16,519: T 17,474.159 ± 10,753.538 vs. C 19,529.143 ± 16,079.418, *p* = 0.490). Further analysis indicated that the ROS levels in PM/DM patients were significantly higher than those in controls (18,477.756 ± 13,574.916 vs. 14,484.191 ± 5703.097, *p* = 0.012).

**Figure 3:**
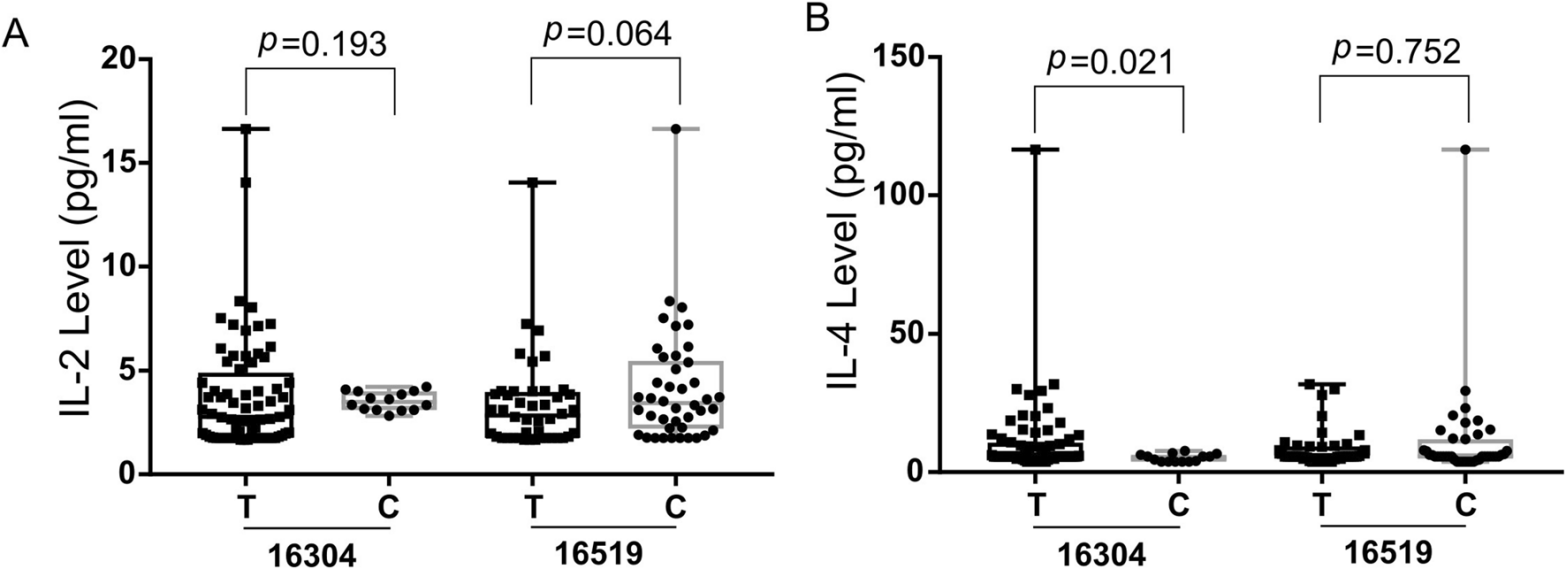
Boxplot of IL-2 **(A)** and IL-4 **(B)** levels in PM/DM associated SNPs. Wilcoxon rank sum test was used to determine significance. *IL-2* interleukin-2, *IL-4* interleukin-4, *PM* polymyositis, *DM* dermatomyositis,* SNP* single nucleotide polymorphisms.

The potential correlation of the levels of cytokines, including interleukin-5 (IL-5), interleukin-13 (IL-13), interferon-γ (IFN-γ), interleukin-2 (IL-2), interleukin-6 (IL-6), interleukin-10 (IL-10), tumor necrosis factor-α (TNF-α), and interleukin-4 (IL-4) and PM/DM risk-associated D-loop SNPs were evaluated using the Wilcoxon rank sum test (Fig. 3, Fig. S2). As shown in Fig. 3, the allele 16304C was significantly associated with lower IL-4 expression (*p* = 0.021), while 16519C had a trend to be associated with higher IL-2 expression (*p* = 0.064). These data suggest that these PM/DM-susceptible SNPs associated with cytokine expression might be involved in PM/DM development.

## Discussion

Mitochondria are semi-autonomous organelles that contain their own DNA and are involved in a series of biological processes, including metabolism, ROS generation, signaling transduction, apoptosis, and calcium homeostasis^9^. The D-loop region correlates with the entire mtDNA replication and transcription; therefore, genetic alterations in this region may affect mitochondrial function through excessive ROS generation and alteration of the immune status^9,20^. Our study found that the alleles 16304C and 16519C affect susceptibility to PM/DM. The allele 16519C showed a high ANA-positive rate, implying that the disease-associated D-loop SNP can result in a failure of self-tolerance and the production of antibodies against human antigens. Allele 16304 in the mutational hotspots of hypervariable segment 1 (HV1) has been identified as an independent prognostic marker for non-Hodgkin lymphoma^21^. In addition, the allele 16519C contributes to gastric cancer carcinogenicity by increasing oxidative DNA damage^22^. Increased ROS generation was observed in PM/DM patients, and elevated ROS directly oxidizes key cellular components, such as proteins and lipids, resulting in oxidative damage of the muscle in IIMs^6^. Chronically elevated ROS levels could impair the function of the electron transport chain to decrease ATP production, thereby weakening the muscles in IIMs^6,23^.

The copy number of mtDNA, which directly correlates with energy reserves, oxidative stress, and changes in the mitochondrial membrane potential, is a promising biomarker of mitochondrial dysfunction^24^. A previous study suggested that a high mtDNA copy number is associated with active bioenergetic function of mitochondria, characterized by increased mtDNA-encoded ND1 gene, succinate-supported oxygen consumption rate, ATP content, and the protein level of mitochondrial transcription factor A^25^. High mtDNA levels in the blood have been reported to be correlated with an increased risk of developing esophageal squamous carcinoma, Keams–Sayre syndrome, and end-stage renal disease^26^. The increased mtDNA copy number may induce mitochondrial dysfunction, thereby initiating the onset of PD/DM. We found that SNPs accumulated excessively in patients with PM/DM, although there is no evidence to support that this difference would lead to a biological effect. We reviewed previous studies on gastric cancer^23^, hepatocellular carcinoma^27^, lung cancer^28^, renal cell carcinoma^29^, lymphoma^30^, rheumatoid arthritis^12^, and systemic lupus erythematosus^13^ and found that SNP accumulation was reported in hepatocellular carcinoma^27^ and SLE^13^, but there were no reports of decreased SNPs in these diseases. Further studies need to be designed to characterize the role of SNP accumulation in disease progression as well as to determine whether SNP amounts positively correlated with mtDNA number is accidental or pathogenic.

As a PM/DM-susceptible SNP associated with ANA expression, 16519C is a valuable biomarker for the disease. It displayed a trend to be associated with higher IL-2 expression (*p* = 0.064), however, we believe that our sample size might limit the potential relationship between IL-2 level and 16519C. Circulating mtDNA leaking out of cells may activate innate immune response to robust type I interferon response^31^, and mtDNA may influence T cells to produce IL-2 via the mitochondrial respiratory complex I-mediated oxidative signaling pathway^32^. IL-2 may act directly on the muscle fibers to synthesize soluble pro-inflammatory mediators, thereby initiating inflammatory myositis^33^. IL-4 appears to be resistant to DM by inhibiting IFN-induced inflammation and enhancing the self-repair ability of the muscles^34^.

Our findings suggest that mitochondrial D-loop SNPs could be potential biomarkers for PM/DM risk and these SNPs associated with cytokine expression may be involved in PM/DM development. Meanwhile, mtDNA copy number-mediated mitochondrial dysfunction may precede the onset of PM/DM.

## Materials and methods

### Tissue specimens and DNA extraction

PM patients (n = 10) and DM (n = 76) patients at the Department of Rheumatology and Immunology, in The Second Hospital of Hebei Medical University, based on the Bohan and Peter criteria between June, 2021 and August, 2021, were recruited in the study^35,36^. The clinical manifestations of PM/DM patients, including age, sex, muscle pain, muscle weakness, heliotrope rash, Gottron’s sign, Shawl’s sign and V-sign, interstitial lung disease (ILD), dysphagia, and laboratory test results such as antinuclear antibody (ANA), Jo-1, CK, LDH, ESR, and CRP, were collected. Simultaneously, 110 age-matched healthy volunteers without immune diseases, cancer, or other chronic diseases were recruited from the Physical Examination Center of The Second Hospital of Hebei Medical University. Total DNA was extracted from blood samples using a genomic DNA extraction kit (Tiangen, Beijing, China). All the procedures were supervised and approved by the Human Tissue Research Committee of The Second Hospital of Hebei Medical University. All experiments were performed in accordance with the relevant guidelines and regulations. Informed consent was obtained from all study participants prior to enrollment.

### Polymerase chain reaction (PCR) amplification and sequence analysis

The primers used in this study were 5'-CCCCATGCTTACAAGCAAGT-3' (nucleotides 16,190–16,209) and reverse 5'-GCTTTGAGGAGGTAAGCTAC-3' (nucleotides 602–583), for the amplification of a 982 bp product in the D-loop. PCR was performed using the DreamTaq Green PCR Master Mix Kit (2 ×) (Thermo Fisher Scientific, Waltham, MA, USA) and purified before sequencing. Cycle sequencing was performed using the BigDye Terminator v3.1 Cycle Sequencing Kit (Life Technologies, Carlsbad, CA, USA), and the products were separated on an ABI PRISM Genetic Analyzer 3100 (Applied Biosystems, Foster City, CA, USA). Polymorphisms were confirmed by repeated analyses of both the strands.

### Measurement of mtDNA copy number

Quantitative real-time polymerase chain reaction (qRT-PCR) analyses were performed to measure the relative mtDNA copy number using an Applied Biosystems 7500 Sequence Detection System (Applied Biosystems). Nuclear DNA and mtDNA were analyzed using the nuclear human β-hemoglobin (HGB) gene and the mitochondrial nicotinamide adenine dinucleotide (NADH) dehydrogenase 1 (ND1) gene^37^. The primers used are listed in Table S2. Genomic DNA (30 ng) was mixed with 3 μL qPCR SYBR Green Mix (5 ×) (GeneCopoeia, Rockville, MD, USA) containing 10 pmol of forward and reverse primers, to make a final volume of 15 μL. Amplification was performed as previously described^38^. The amplification specificity was validated by using melting curve analysis. The copy number of mtDNA in each specimen was estimated using the 2^-ΔΔCt^ relative expression formula (ΔCt = Ct ND1 − Ct HGB). The measurements were repeated twice, with a sample of unchanged DNA placed in the same well position of the tray as a reference.

### ROS measurement

The BBOXiProbe^®^ Plasma Active Oxygen Detection Kit (BestBio Technology, Shanghai, China) was used to determine the ROS levels. Briefly, 100 μL serum was incubated with 10 μL of O12 probe for 30 min at 37 °C. ROS levels were measured using a fluorescence microplate reader (BIOTEK, Winooski, VT, USA) with an excitation wavelength of 488 nm and an emission wavelength of 520 nm.

### Cytokines measurement

The Human TH1/TH2 Panel (8-Plex) with Filter Plate V02 was used to measure the concentrations of cytokines, including IL-5, IL-13, interferon-γ (IFN-γ), IL-2, IL-6, IL-10, TNF-α, and IL-4 (BioLegend, San Diego, CA). The 25 μL serum sample (twofold diluted with assay buffer) was incubated with 25 μL antibody and mixed with beads for 1 h at room temperature in the dark, shaking at approximately 800 rpm. Following this, 25 µL streptavidin–phycoerythrin (SA-PE) was added to each medium and shaken at approximately 800 rpm, for 30 min at room temperature in the dark. The PE fluorescence signal of the analyte-specific bead region was quantified using a MACSQuant Analyzer 10 flow cytometer (Miltenyi Biotec, Bergisch Gladbach, Germany), and the concentration of the specific analyte was determined using LEGENDplexTM Data Analysis Software (BioLegend, San Diego, CA).

### Statistical analysis

For continuous variables, Student's t-test was used for the independent samples, and the Wilcoxon rank sum test was used if the assumptions of normality for the t-tests were not fulfilled. The chi-square test or Fisher's exact test was used to assess the independence of categorical variables in the contingency table. Pearson's correlation test was used to assess the relationship between the variables, and Spearman’s correlation test was used if the assumptions of normality were not fulfilled. All statistical analyses were performed using the SPSS software (version 19.0; IBM Corporation, Armonk, NY, USA). Statistical significance was set at *p* < 0.05.

## Supplementary Information

Supplementary Information.

## Data Availability

Research data supporting the results of this paper will be provided by corresponding author at reasonable request.
