# Supplementary material for: The SNPs of mitochondrial DNA displacement loop region and mitochondrial DNA copy number associated with risk of polymyositis and dermatomyositis
Source: Sci Rep. 2022 Apr 7;12:5903. doi: 10.1038/s41598-022-09943-x (PMC8990067; doi:10.1038/s41598-022-09943-x)
Supplement: Supplementary file 1 — Supplementary Information. [file 41598_2022_9943_MOESM1_ESM.docx]

**The SNPs of mitochondrial DNA displacement loop region and** **mitochondrial DNA copy number associated with risk of polymyositis and dermatomyositis**

Yufei Zhao^1^, Chenxing Peng^2^, Ruixue Lai^1^, Jingjing Zhang^1^, Xiaoyun Zhang^1^, Zhanjun Guo^1,*^

^1^Department of Immunology and Rheumatology, The Fourth Hospital of Hebei Medical University, Shijiazhuang, Hebei 050011, P.R. China

^2^Department of Immunology and Rheumatology, The Second Hospital of Hebei Medical University, Shijiazhuang, Hebei 050000, P.R. China

***Correspondence author:**

Zhanjun Guo, M.D. and Ph.D.

Department of Immunology and Rheumatology, The Fourth Hospital of Hebei Medical University, 12 Jiankang Road, Shijiazhuang 050011, P.R. China.

Tel: + 86 311 8609 5342. Fax: + 86 311 8609 5237.

1. mail: [zjguo5886@aliyun.com](mailto:zjguo5886@aliyun.com).


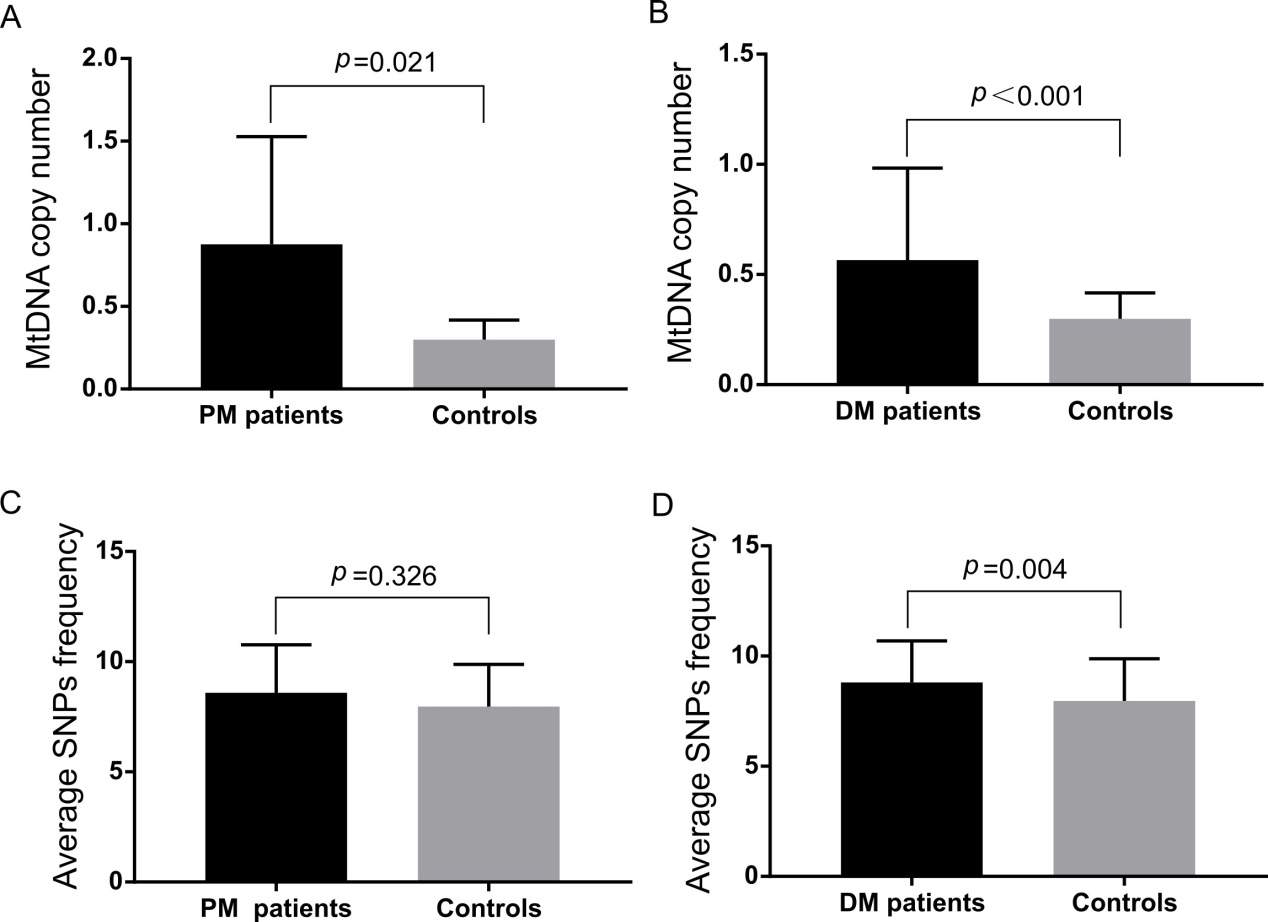


**Figure S1.** MtDNA copy numbers and average SNPs frequency in subgroups of PM/DM patients and controls. (A) MtDNA copy number in PM patients and controls. (B) MtDNA copy number in DM patients and controls. (C) The average frequency of SNPs in PM patients and controls. (D) The average frequency of SNPs in DM patients and controls.

MtDNA: mitochondrial DNA; PM: polymyositis; DM: dermatomyositis; SNP: single nucleotide polymorphisms.


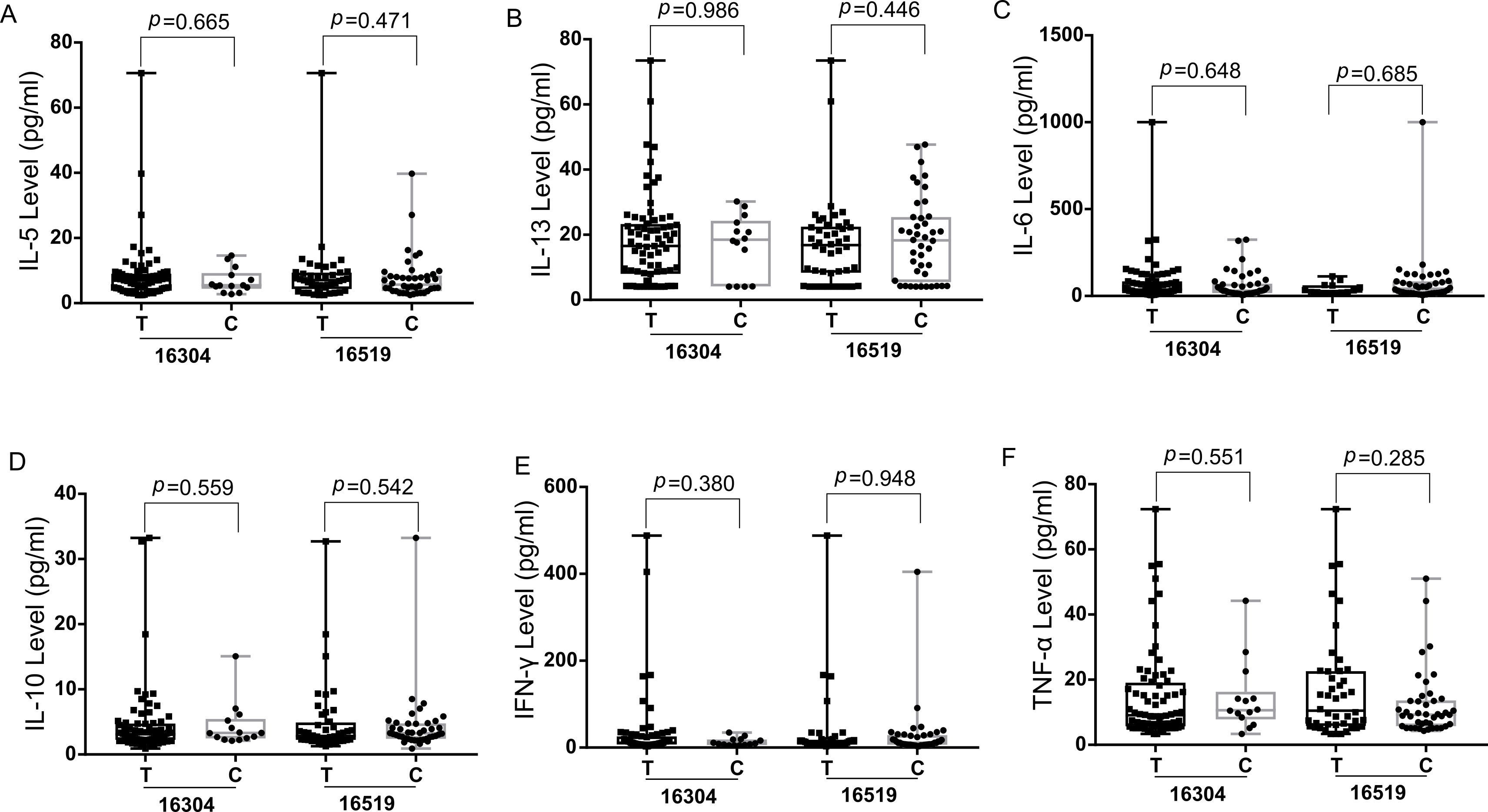


**Figure S2.** Boxplot of IL-5(A), IL-13(B), IL-6(C), IL-10(D), IFN-γ(E) and TNF-α(F) levels in PM/DM risk-associated SNPs. Wilcoxon rank sum test was used to determine significance.

IL-5: Interleukin-5; IL-13: Interleukin-13; IL-6: Interleukin-6; IL-10: Interleukin-10; IFN-γ: interferon-γ; TNF-α: Tumor Necrosis Factor-α; PM: polymyositis; DM: dermatomyositis; SNP: single nucleotide polymorphisms.

| Nucleotide | PM patients (n=10) | DM patients (n=76) | Controls (n=110) | χ² | *p* | OR |
| --- | --- | --- | --- | --- | --- | --- |
|  |  |  |  |  |  |  |
|  |  |  |  |  |  |  |
| 16304T/C | 9/1 (90% / 10%) | -- | 102/8 (92.7% / 7.3%) | -* | 0.556 | 1.417 |
|  | -- | 63/13 (82.9% / 17.1%) |  | 4.339 | 0.037 | 0.380 |
| 16519T/C | 4/6 (40% / 60%) | -- | 72/38 (65.5% / 34.5%) | 1.579 | 0.209 | 2.842 |
|  | -- | 40/36 (52.6% / 47.4%) |  | 3.085 | 0.079 | 0.586 |
| **Table S1.** Subgroups analysis for PM/DM susceptible single nucleotide polymorphisms in the D-loop.  * Fisher's exact test; PM: polymyositis; DM: dermatomyositis;  χ²:Chi-square; OR: Odds ratio. | | | | | | |

|  | |
| --- | --- |
| HGB forward | 5' GTGCACCTGACTCCTGAGGAGA 3' |
| HGB reverse | 5' CCTTGATACCAACCTGCCCAG 3' |
| ND1 forward | 5'-CCCTAAAACCCGCCACATCT-3' |
| ND1 reverse | 5'-GAGCGATGGTGAGAGCTAAGGT-3' |
| **Table S2.** Primers used in copynumber analysis.  HGB: human β-haemoglobin; ND1: nicotinamide adenine dinucleotide dehydrogenase 1. | |
|  |  |
